# Rethinking Parkinson's disease: could dopamine reduction therapy have clinical utility?

**DOI:** 10.1007/s00415-024-12526-7

**Published:** 2024-06-21

**Authors:** Jonathan Sackner-Bernstein

**Affiliations:** Right Brain Bio, Inc, Pleasantville, NY 10570 USA

**Keywords:** Parkinson’s, Dopamine, Tyrosine hydroxylase, Synucleinopathy

## Abstract

Following reports of low striatal dopamine content in Parkinson’s disease, levodopa was shown to rapidly reverse hypokinesis, establishing the model of disease as one of dopamine deficiency. Dopaminergic therapy became standard of care, yet it failed to reverse the disease, suggesting the understanding of disease was incomplete. The literature suggests the potential for toxicity of dopamine and its metabolites, perhaps more relevant given the recent evidence for elevated cytosolic dopamine levels in the dopaminergic neurons of people with Parkinson’s. To understand the relevance of these data, multiple investigations are reviewed that tested dopamine reduction therapy as an alternative to dopaminergic agents. The data from use of an inhibitor of dopamine synthesis in experimental models suggest that such an approach could reverse disease pathology, which suggests that cytosolic dopamine excess is a primary driver of disease. These data support clinical investigation of dopamine reduction therapy for Parkinson’s disease. Doing so will determine whether these experimental models are predictive and this treatment strategy is worth pursuing further. If clinical data are positive, it could warrant reconsideration of our disease model and treatment strategies, including a shift from dopaminergic to dopamine reduction treatment of the disease.

## Introduction

In the 1960s, investigations established that Parkinson’s patients suffered from dopamine depletion [1–4], subsequently recognized to be driven in part by loss of dopaminergic neurons [5]. Unfortunately, the dopaminergic therapies developed to address this deficiency are incapable of slowing, halting, or reversing disease progression [6, 7], suggesting that dopamine deficiency represents an incomplete understanding of the disease mechanism. Several models contemplate this shortcoming, including consideration of the role of dopamine and its metabolites [6–10].

The cautionary view that these dopaminergic therapies could be toxic prompted recommendations ranging from postponing initiation of therapy to treatment holidays [11–13]. These concerns were not supported by data from multi-center, randomized, placebo-controlled trials [14–16], which also undermined concern for a role of dopamine driven disease progression. If dopamine were a disease driver, it could be argued that given the extremely low levels of dopamine in human striatum [1–4, 17], realizing pharmacologic levels with levodopa-based therapies would be expected to reveal clinical toxicity.

**Fig. 1:**
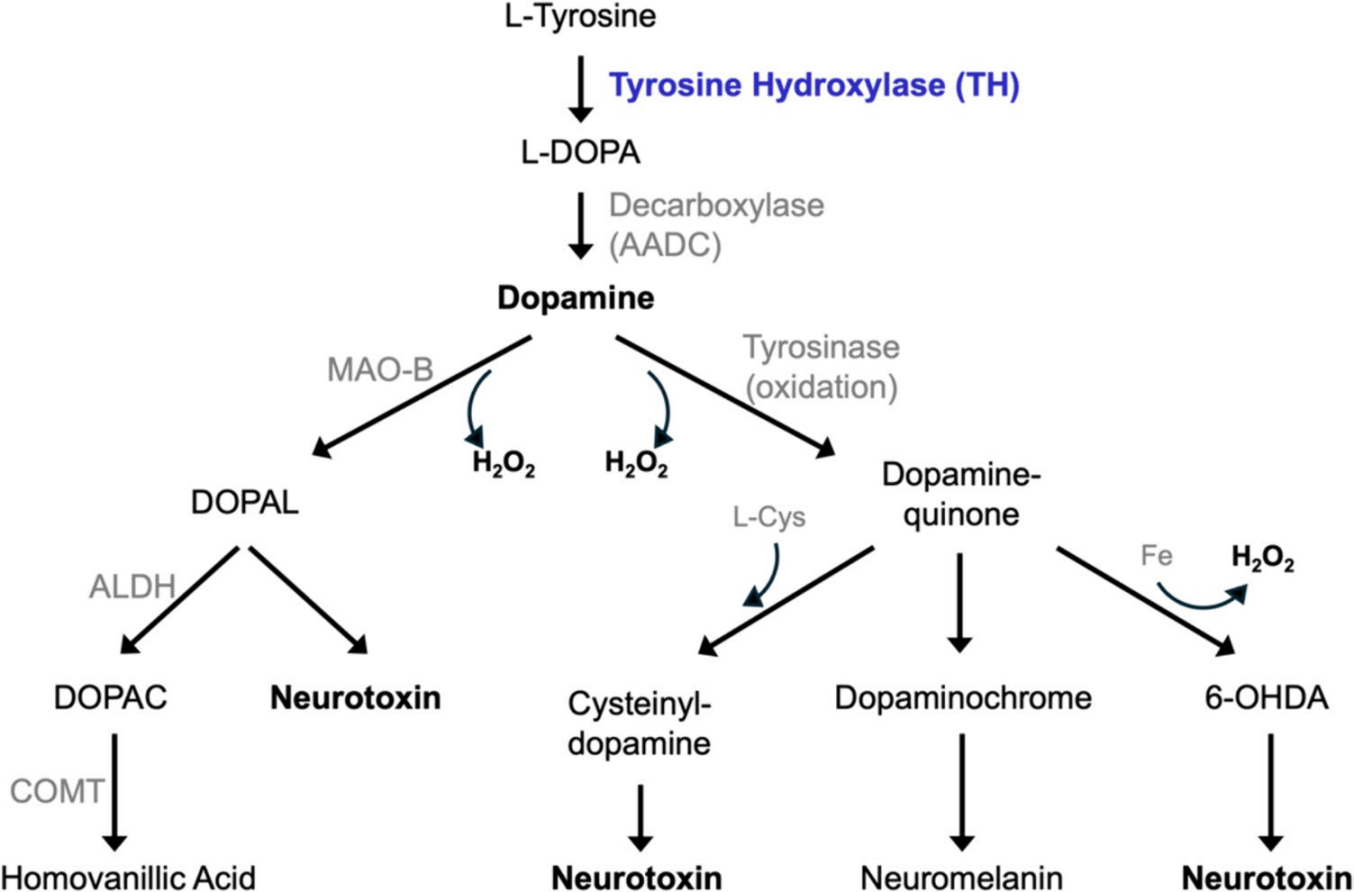
Metabolic pathways of dopamine

However, two lines of reasoning suggest the concerns about dopaminergic pathology should not be dismissed. First, low levels of dopamine reported in Parkinson’s patients may be misleading because they are tissue concentrations rather than neuron-based measures [1–4, 17]. Dopamine-related toxicity is determined by the amount of dopamine free within the cytosol of the dopaminergic neurons, where it is oxidized and metabolized into toxic byproducts (Fig. 1). Tissue concentrations represent a combination of intra- and extracellular fluids, with the intracellular compartment containing a combination of dopaminergic neurons and other cells and are not representative of what the dopaminergic neurons experience. In this context, low cytosolic dopamine levels would be required to support the current model of disease. But despite studies that suggest cytosolic levels could be elevated [18] as appears to be its turnover [19], the standard of care does not consider dopamine toxicity as an important risk [6, 7]. Second, studies in multiple preclinical models show that dopamine reduction therapy via tyrosine hydroxylase inhibition reverses disease pathology. In models ranging from drosophila [20] to rodents [21–23], including cell culture studies of multiple types [20, 24–27], inhibition of tyrosine hydroxylase reversed measures of disease pathology including oxidative stress, alpha-synuclein deposition, neuron loss, and impaired movement (Table 1).

**Table 1:** Effect of metyrosine in models of Parkinson’s disease

Model	Publication	Findings
Amphetamine toxicity	Axt 1990 [21]	Metyrosine protects against methamphetamine associated toxicity
MPP + mice	Choi 2015 [22]	Metyrosine improves neuron survival after MPTP toxin exposure
PARKIN mutant rat	Dong 2003 [23]	Metyrosine preserves neurons and axonal fiber density
SH-SH5Y^a^ overexpressing alpha-synuclein	Xu 1999 [24]	Metyrosine blocks apoptosis
SH-SH5Y^a^ overexpressing alpha-synuclein	Xilouri 2009 [25]	Metyrosine reverses dysfunctional lysosomal proteolysis
SH-SY5Y^a^	Zhou 2022 [20]	Metyrosine reduces Fe^2+^ or Fe^3+^ induced oxidative stress
PC-12^b^	Kitazawa 2002 [27]	Metyrosine blocks mitochondrial toxin (MMT) mediated apoptosis
Human fetal dopaminergic cells	Xu 1999 [24]	Metyrosine blocks alpha-synuclein triggered apoptosis
Human iPSC cultures (normal and DJ-1)	Burbulla 2017 [26]	Metyrosine reduces dopamine oxidation and blocks alpha-synuclein deposition
Human mutant LRRK2 transfected drosophila (G2019S)	Zhou 2022 [20]	Metyrosine reverses hypokinesis and improves survival

^a^SH-SY5Y: neuroblastoma cell line

^b^PC-12: rat pheochromocytoma cell line

It is therefore plausible that dopamine and its metabolites serve a central role in the pathology of Parkinson’s disease and these observations support testing of dopamine reduction therapy in the clinic. Three factors undermine the current model of disease: first, dopamine levels in the cytosol are elevated in Parkinson’s disease, meaning it could be a factor, second, dopamine-related toxicity explains much of the understanding of disease, suggesting at least an association of the molecule with disease progression and third, multiple laboratory models show that dopamine reduction therapy can reverse pathology, supporting a causative role of dopamine and its metabolites in Parkinson’s disease. While possible to reduce cytosolic dopamine by targeting the vesicles or vesicular monoamine transporter 2 (VMAT2), the dopamine-excess toxicity model of disease can be tested immediately through study of dopamine reduction therapy in the clinic using an FDA-approved, and therefore, repurposed drug to inhibit tyrosine hydroxylase [28].

## Dopamine excess in Parkinson’s disease

The basis of our understanding of dopamine’s function in the central nervous system includes: that dopamine is a neurotransmitter [29], that dopamine plays a critical role in movement, and therefore, Parkinson’s disease and Parkinsonian states [30, 31] and that dopamine’s function within neurons is tightly controlled [32].

Dopamine metabolites can exert toxic effects, including via oxidized dopamine and hydrogen peroxide generation as well as aldehyde and quinone metabolites. Normally, production of these toxins is prevented by sequestration of dopamine within intracellular vesicles [8, 33, 34], which also serve as the reservoir for release of dopamine into the synapse for neurotransmission. However, vesicular storage has been shown to be impaired in both laboratory [34] and clinical settings [33], which is manifested as a functional impairment and a decrease in number on these intracellular structures [33]. The vesicular dysfunction inherent to Parkinson’s disease shifts dopamine into the cytosol, where toxic metabolites are produced. The level of free, cytosolic dopamine in the dopaminergic neurons must be investigated to define how these cells are affected by the disease. It is possible to estimate the relative content of free, cytosolic dopamine in dopaminergic neurons of people with Parkinson’s disease compared to those without the disease. An algebraic approach permits estimation of dopaminergic neuron dopamine content by scaling the tissue dopamine level by the number of neurons or axons and then to the number of vesicles [17].

**Fig. 2:**
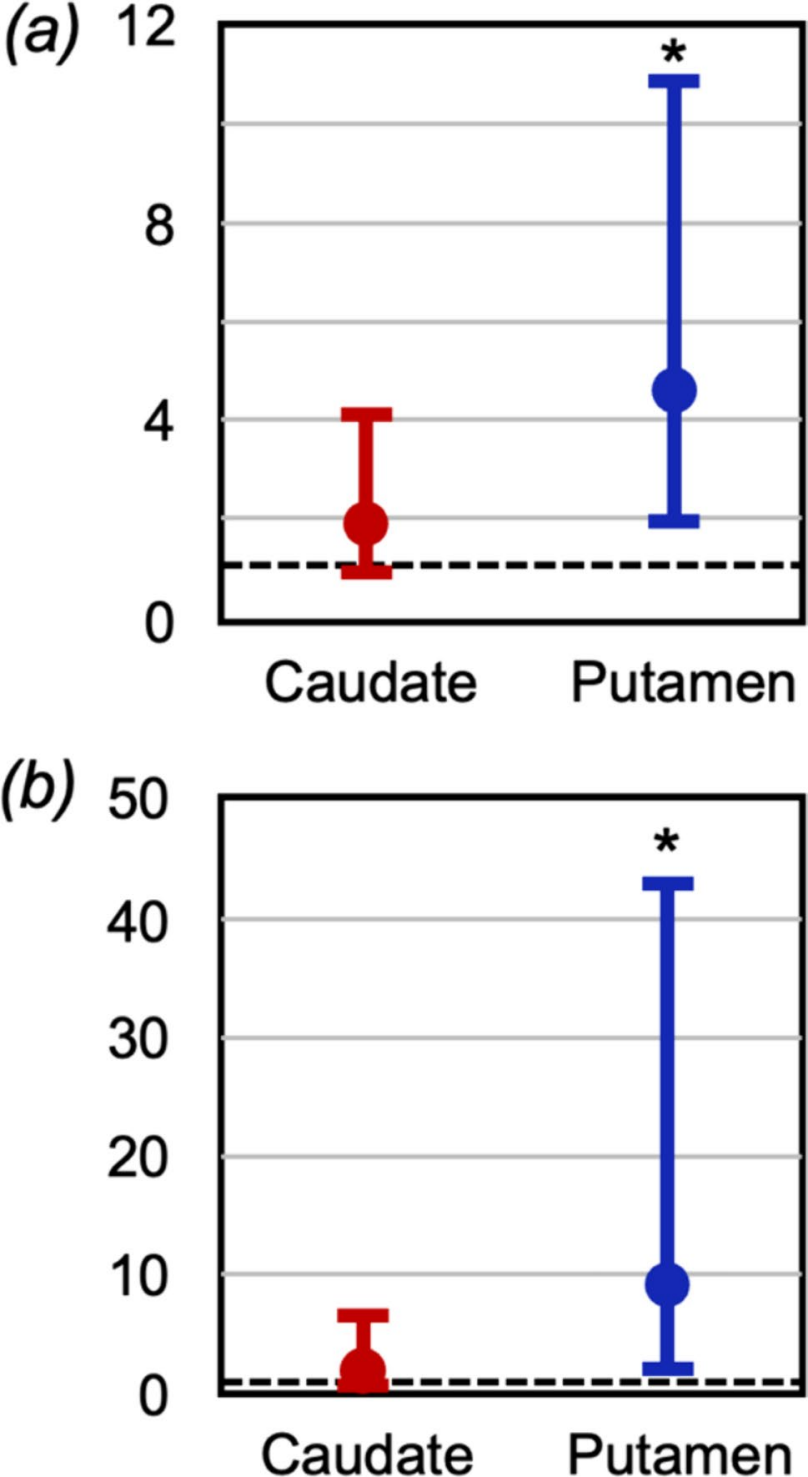
Estimates of the ratio of cytosolic dopamine in people with Parkinson’s compared to age-matched controls, as calculated by step-wise meta-analysis [17]. (**a**) the ratio in the caudate is 1.87 (95% CI 0.85, 4.11, *p* = 0.12) and in the putamen is 4.61 (95% CI 1.95, 10.9, *p* = 0.001). (**b**) when restricted to those people with tissue dopamine concentrations measured in absence of dopaminergic therapy, the ratio in the caudate is 1.72 (95% CI 0.46, 6.43, *p* = 0.42) and in the putamen is 9.07 (95% CI 1.92, 42.9, *p* = 0.005)

Ideally, such an approach would be based on samples from one population. Without such a dataset, the calculation was performed using data from distinct clinical populations. Via a step-wise meta-analysis, the ratio of cytosolic dopamine in Parkinson’s tissue compared to non-Parkinson’s tissue was 1.87 (95% CI 0.85, 4.11, *p* = 0.12) in the caudate and 4.61 (95% CI 1.95, 10.91, *p* = 0.001) in the putamen (Fig. 2a). Limiting the calculations to include only those with dopamine tissue levels in the absence of dopaminergic therapies revealed ratios of 1.72 (95% CI 0.46, 6.43, *p* = 0.42) in the caudate and 9.07 (95% CI 1.92, 42.88, *p* = 0.005) in the putamen, (Fig. 2b) qualitatively similar to the overall population [17].

This contrarian result that Parkinson’s disease features increased dopamine exposure for the dopaminergic neurons is supported by prior studies of brains from people with Parkinson’s [18, 19]. Potential causes for increased dopamine in the neuronal cytosol include decreased breakdown, increased production, or shift from the vesicular to cytosolic compartment. It does not appear that dopamine degradation is meaningfully reduced [35]. Tyrosine hydroxylase activity in human striatal tissue homogenates can be measured based on l-3,4-dihydroxyphenylalanine (L-DOPA) synthesis per mg of tyrosine hydroxylase. The enzymatic activity scaled to the amount of protein present showed markedly elevated synthetic activity in the caudate (~ 4.0x) and putamen (~ 2.7×) of people with Parkinson’s compared to those without the disease [18]. These data are consistent with the reanalysis [19] of data from Bernheimer and Hornykiewicz [36] showing dopamine turnover was ~ 13 × higher in Parkinson’s. Dopamine uptake by neuronal vesicles was significantly impaired in both caudate and putamen of people with Parkinson’s [33]. These findings support the calculation showing that dopamine levels are estimated to be elevated in the cytosol of dopaminergic neurons of people with Parkinson’s [17].

**Fig. 3:**
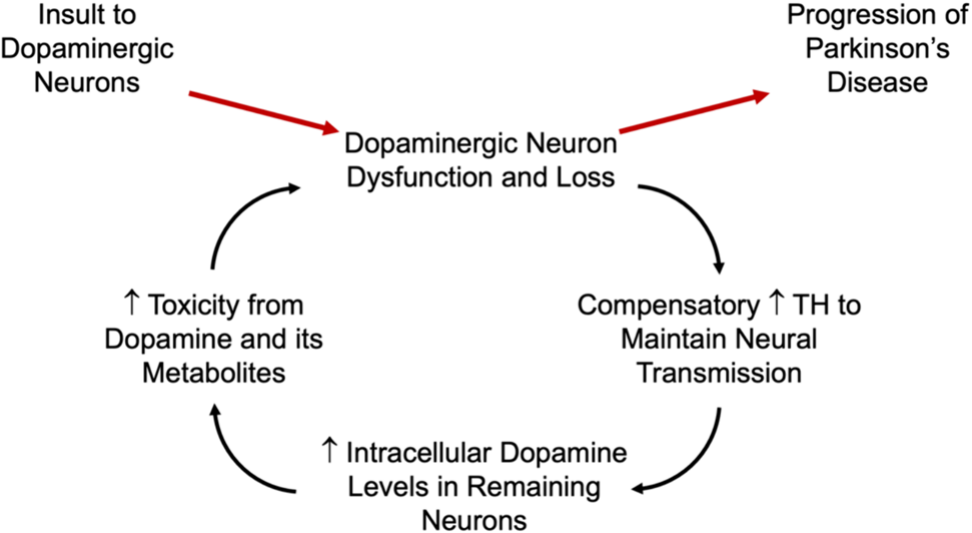
Dopamine-related toxicity model of Parkinson’s disease onset and progression

Studies of acute striatal injury also suggest a potential role for dopamine-related toxicity in the onset of disease (Fig. 3). Unilateral striatal injection of 6-hydroxydopamine in rats increases specific activity of tyrosine hydroxylase locally but not in the contralateral striatum [37], as if it were a compensatory response to the local toxic injury within the striatum. Similarly, when newborn pigs were exposed to mild hypoxia, dopaminergic synthesis rose and continued to do so after cessation of hypoxia, to ~ 140% of baseline level [38], consistent with a compensatory response to the ischemic injury to the striatum. Thus, two types of acute striatal injuries lead to increased dopamine synthesis, suggesting the possibility of its role as an early contributor to disease.

Dopamine, directly and/or through its metabolites (Fig. 1), is shown in multiple laboratory studies to be associated with dopaminergic cell death [34, 39–42]. The literature is reviewed to learn whether a causal relationship is plausible between dopamine-related toxicity and Parkinson’s disease.

## Dopamine toxicity in Parkinson’s disease

### Oxidative stress

Dopamine metabolism produces hydrogen peroxide, reactive oxygen species [9], and oxidized dopamine [33]. Alpha-synuclein oligomers that form in the presence of dopamine and its metabolites further increase oxidative stress, with the oligomer and protofibrillar forms favored by dopamine over fibrils markedly increasing hydrogen peroxide (H_2_O_2_) and reactive oxygen species (ROS) production [43]. Rat striatum injected with dopamine formed cysteinyl-dopamine and cysteinyl-3,4-dihydroxyphenylacetic acid (DOPAC), adducts that relate to oxidative stress and which caused loss of neurons within days [44]. Calcium entry into murine dopaminergic neurons is critical to pacemaking function and triggers oxidative stress [45]. The normal process of ATP-dependent removal of calcium from mitochondria would be impaired in Parkinson’s disease as discussed in the context of mitochondrial dysfunction in Parkinson’s disease. In chronic cultures of human iPSCs from sporadic and DJ-1 mutant homozygous Parkinson’s disease, the oxidation level increased over 150–180 days and was blocked by metyrosine, an inhibitor of tyrosine hydroxylase [26].

### Neuron viability

Dopamine is toxic to neurons [39, 40]. In culture, cell death is related to dopamine levels [46]. In mice overexpressing the dopamine transporter (DAT), increased presynaptic dopamine uptake led to reduced VMAT2 function, cytosolic buildup of dopamine metabolites, and eventually neuron death and motor dysfunction [40]. When alpha-synuclein couples to human DAT in cell culture, presynaptic dopamine overload follows, triggering apoptosis [47]. While this neuronal loss is fundamental to Parkinson’s disease, evidence suggests that dopaminergic neurodegeneration starts with the axons [34, 48]. In contrast, multiple approaches that reduce cytosolic dopamine improve neuronal survival. Aromatic amino acid decarboxylase (AADC) inhibition reduces dopamine in cell culture, preserving neurons [46]. Overexpressing VMAT2 shifts dopamine from the cytosol to the vesicles, also preserving neurons [46]. Inhibition of presynaptic dopamine reuptake by addition of the DAT blocker nomifensine preserves neurons [41].

### Mitochondrial function

As one of the most densely arborized axonal networks, the dopaminergic innervation of the striatum requires a correspondingly high density of mitochondria. In human striatal tissue, Parkinson’s patients can show almost no complex I activity and markedly reduced flavoprotein levels [49]. In rat brain mitochondria, the quinone forms of dopamine and DOPAC have been shown to inhibit complex I function in dose-related fashion [50] and dopamine–quinone-impaired mitochondrial permeability transition pore (mPTP) function, which led to mitochondrial swelling [51]. Abnormal mPTP function was shown as well in A53T expressing rat cortical neurons driven by oxidized dopamine, which led to caspase mediated apoptosis [52]. Cells cultured in dopamine had increased H_2_O_2_, reduced mitochondrial inner membrane potential, and decreased ATP production [53]. The relevance of intact mitophagy is supported by evidence that soluble PARKIN levels decreased with increased dopamine levels in MES23.5 dopaminergic cells [54]. In parallel, PARKIN was present in a larger proportion in its insoluble form in the caudate of people with sporadic Parkinson’s disease [54]. Insoluble PARKIN does not permit its normal role in mitophagy. In a striatal toxin model (methylcyclopentadienyl manganese tricarbonyl), activation of mitochondrial caspase-3-mediated apoptosis is blocked by metyrosine [27]. Mitochondrial membrane cardiolipin triggers alpha-synuclein oligomerization, most pronounced with its A53T form [52]. The interrelationships between diverse mechanistic contributors to Parkinson’s disease were shown in human iPSC studies, showing loss of mitochondrial potential, increased oxidative stress, reduced complex I activity, increased mPTP opening, and cell loss [52]. With the loss of mitochondrial function, presynaptic dopamine terminals suffer from this energy crisis.

### Alpha-synuclein

Binding of dopamine with alpha-synuclein occurs in specific ratios [55] suggesting a teleologic basis for binding of dopamine with alpha-synuclein. Alpha-synuclein oligomerization is stimulated by dopamine [56, 57], 3,4-dihydroxyphenylacetaldehyde (DOPAL) [58, 59] and dopamine–quinone [60], with both dopamine and DOPAL doing so in a time- and concentration-dependent manner [57–59]. Oligomeric and protofibrillar forms are favored as both dopamine and DOPAL inhibit fibril formation [56, 57]. In the absence of dopamine, no oligomers are formed [56] and low levels of dopamine bind dopamine and its metabolites as if to block toxic effects [61].

Alpha-synuclein can increase cytosolic dopamine. Overexposure to alpha-synuclein damages vesicles which then leak dopamine into the cytoplasm [46]. Further, alpha-synuclein binds to human DAT leading to increased presynaptic dopamine uptake and presynaptic dopamine overload [47]. Control of dopamine synthesis in Parkinson’s disease is not clear. Alpha-synuclein is reported to inhibit tyrosine hydroxylase [62, 63] as well as increase its activity [46]. And while alpha-synuclein exerts toxic effects, low levels of alpha-synuclein protect SH-SY5Y cells by binding dopamine and its metabolites [61]. In chronic cultures of human-induced pluripotent stem cells (iPSCs), metyrosine is shown to reduce alpha-synuclein deposition while L-DOPA increases alpha-synuclein deposition [26]. And metyrosine blocks alpha-synuclein-mediated apoptosis [24].

There do not appear to be simple explanations for the contradictory data regarding the effect of alpha-synuclein on tyrosine hydroxylase activity and balance between toxic and neuroprotective effects [46, 62, 63]. Without obvious methodologic shortcomings in these studies, explanations include the possibility that not all the models are equally relevant to clinical Parkinson’s disease as well as that unmeasured variables, essentially unknown factors, are not measured which would provide additional clarity. Resolving these issues is beyond the scope of this review.

### VMAT2 and vesicles

In neurons from people with Parkinson’s disease, dopamine uptake and storage of dopamine are impaired [33], which shifts dopamine from the vesicles to into the cytosol. In murine neurons, DOPAL triggers alpha-synuclein oligomerization [64], which decreases vesicle motility and the processes leading to release of dopamine into the synapse to complete neurotransmission. The oligomerization induced by DOPAL damages vesicles [64] allows for proton efflux and dopamine leakage into the cytosol [64]. This dopamine is metabolized to DOPAL which amplifies the cycle of dysfunction. Vesicular leakage is seen in the presence of wild type, A30P or A53T forms of alpha-synuclein [46]. Reduced vesicular motility and uptake could be driven by the mitochondrial dysfunction in Parkinson’s disease, given the vesicular need for ATP/GTP.

### Inflammation

Inflammation is evident in Parkinson’s disease. TNF-alpha levels are elevated in the striatum and cerebrospinal fluid of people with Parkinson’s patients [65]. In parallel, serum levels are elevated [66]. IgG from peripheral blood of Parkinson’s patients triggers TNF-alpha release in mouse microglia [67]. TNF-alpha modulates tyrosine hydroxylase activity. When peripheral monocytes from healthy elderly subjects are exposed to TNF-alpha, tyrosine hydroxylase level increases as observed in monocytes from people with Parkinson’s disease [68]. Dopamine’s effects on neuroinflammation are not as clearly defined. Dopamine is a potent chemoattractant with effects on microglia similar to the effect of MCP-1 [69]. Dopamine–quinone triggers TNF-alpha release [67]. While a connection between dopamine and neuroinflammation is supported by the literature, whether dopamine is a trigger for neuroinflammation is not yet established.

### Implications

The roles of dopamine and its metabolites in Parkinson’s disease are well documented. However, the relative contribution of these mechanisms to disease onset and progression is difficult to define, particularly given the problems of studying the pathophysiology of Parkinson’s in people with the disease to fully elucidate the cellular, molecular, and genetic triggers and drivers of the disease. With the literature showing that a causal relationship of dopamine to Parkinson’s disease pathogenesis is plausible, the next step is testing the effects of dopamine reduction therapy in models of disease.

## Dopamine reduction therapy in Parkinson’s

Randomized, placebo-controlled, double-blind clinical trials show that dopaminergic therapies improve clinical status in Parkinson’s disease [14–16]. However, none of these trials extended past 40 weeks and no therapy is shown to affect disease progression [14–16]. Given the recent evidence for cytosolic dopamine excess in Parkinson’s and the multiple pathways for dopamine-related toxicity, the effects of dopamine reduction therapy warrant consideration.

While the targets for reversing cytosolic dopamine excess include reversing vesicle dysfunction and inhibition of dopamine synthesis, only the latter can be addressed by an FDA-approved drug. Using a repurposed drug allows for more rapid testing in clinical trials and less concern about off-target toxicity. The drug metyrosine (alpha-methyl-p-tyrosine) is a reversible inhibitor of tyrosine hydroxylase that crosses the blood–brain barrier and which does not impact serotonin synthesis [70, 71].

Several models of Parkinson’s disease and/or dopamine excess show the effects of metyrosine (Table 1). Metyrosine preserved dopaminergic function following an insult with methamphetamine-induced cytosolic dopamine release [21].

Two rodent models show preservation of striatal architecture. In the murine MPP + model, dopaminergic neuron survival was preserved by metyrosine [22] and in a rat PARKIN mutant strain, fiber density was preserved in parallel with improved neuron survival [23].

Metyrosine’s effects have also been studied in two engineered cell lines. The neuroblastoma cell line SH-SY5Y was used by three laboratories. Cells overexpressing alpha-synuclein—wild type, A30P, or A53T—underwent apoptosis and this was blocked by metyrosine [24]. Cells overexpressing wild type alpha-synuclein suffered lysosomal proteolysis and nuclear loss that was reversed with metyrosine [25]. Cell survival was preserved by metyrosine when these SH-SY5Y cells were subjected to oxidative stress from ionized iron [20]. The rat pheochromocytoma cell line PC-12 when cultured with a mitochondrial toxin MMT(methylcyclopentadienyl manganese tricarbonyl) led to an increase in caspase activity, and the resulting apoptosis as reflected by DNA fragmentation, which was blunted by metyrosine [27].

Similarly, human-sourced cell cultures have been employed to study the effects of metyrosine. When human fetal dopaminergic cells were engineered to overexpress alpha-synuclein, wild type, A30P, or A53T, the rate of apoptosis was markedly reduced with metyrosine therapy [24]. Human-induced pluripotent stem cells (iPSCs) were generated from people with sporadic and DJ-1 homozygous Parkinson’s disease and an age-matched control. When grown in culture for 50–180 days, metyrosine reduced oxidized dopamine levels and alpha-synuclein deposition. These cell lines also were studied with exposure to L-DOPA, during which alpha-synuclein deposition was increased in DJ-1 but not in wild type Parkinson’s lines, along with reduced neuron survival in DJ-1 homozygous dopaminergic iPSCs [26]. Drosophila transfected with human mutant LRRK2 gene (G2019S) developed hypokinesis similar to a Parkinson’s phenotype, which was reversed with metyrosine therapy in parallel with improved survival [20].

With the benefits of metyrosine being reported in a range of in vitro and in vivo models of Parkinson’s/alpha synucleinopathy, clinical testing of dopamine reduction therapy warrants consideration [28]. Preclinical and clinical pharmacology identify doses of metyrosine sufficiently low that tolerability appears more likely [74–76]. Clinical testing would need to focus on the initiation of therapy at low doses before use of the doses currently available for the marketed product because of obvious concerns in making patients worse [28]. And while target populations would start with the treatment-naïve, understanding the utility of dopamine reduction relative to dopaminergic agents would require additional clinical trials.

The clinical impact of dopamine reduction therapy is possible to conjecture based on the uniformity of impact seen in clinical trials, assuming that the preclinical and clinical pharmacology data correctly identify a dose regimen to assure tolerability as this dopamine reduction therapy is introduced [74–76]. These pharmacological data indicate that use of the high dose available commercially is more likely to precipitate clinical worsening and therefore should not be a consideration.

If Hirsch’s hypothesis of “suffering” neurons is true [77], then reducing dopamine could reverse the disease pathology in these dysfunctional neurons and preserve the functional neurons, potentially restoring movement and cognition in the long term. With protection from dopamine and its metabolites, perhaps disease progression can be halted or even reversed. Would further studies in other experimental models be reasonable next step? Perhaps we could learn more about the science, but given the inability for any single model to reliably predict clinical effects, it seems reasonable to move directly to clinical studies, particularly with a repurposed drug available [28]. Such clinical trials will define the place of dopamine reduction relative to dopaminergic agents in the treatment of disease and determine whether excess cytosolic dopamine is a primary driver of disease.

## Summary

From the viewpoint of dopaminergic neurons, Parkinson’s disease is one of dopamine excess as evidenced by increased levels of cytosolic dopamine in both caudate and putamen [17], consistent with studies showing increase in homospecific tyrosine hydroxylase activity and increased dopamine turnover in people with Parkinson’s disease [18, 19]. The toxicity from dopamine and its metabolites appears to plausibly contribute to several pathologic mechanisms of Parkinson’s, including increased oxidative stress [26, 43–45], mitochondrial dysfunction [49–54], alpha-synuclein oligomerization [47, 56–59], vesicle dysfunction[46, 64], and neuronal death [34, 39–42]. Dopamine reduction therapy is shown in multiple models of Parkinson’s disease to reverse pathology [20–24, 26, 27], and pharmacological data with metyrosine provides a rational dosing regimen [74–76] to test whether Parkinson’s is a disease of dopamine excess as proposed herein, or one of dopamine deficiency as is the currently accepted model of disease. Clinical trials offer hope to identify a new treatment strategy for Parkinson’s disease, and if the data were to support dopamine reduction therapy, then in parallel, a revision of the model of disease would be warranted.
